# Assessment of Rat Sciatic Nerve Using Diffusion-Tensor Imaging With Readout-Segmented Echo Planar Imaging

**DOI:** 10.3389/fnins.2022.938674

**Published:** 2022-06-23

**Authors:** Yueyao Chen, Zhongxian Pan, Fanqi Meng, Qian Xu, Leyu Huang, Xuejia Pu, Xuewen Yu, Yanglei Wu, Hanqing Lyu, Xiaofeng Lin

**Affiliations:** ^1^Department of Radiology, Shenzhen Traditional Chinese Medicine Hospital, The Fourth Clinical Medical College of Guangzhou University of Chinese Medicine, Shenzhen, China; ^2^Department of Pathology, Shenzhen Traditional Chinese Medicine Hospital, The Fourth Clinical Medical College of Guangzhou University of Chinese Medicine, Shenzhen, China; ^3^Siemens Healthineers, Beijing, China; ^4^Department of Nuclear Medicine, The Seventh Affiliated Hospital, Sun Yat-sen University, Shenzhen, China

**Keywords:** magnetic resonance imaging, diffusion tensor imaging, peripheral nerve, readout-segmented echo-planar imaging, sciatic nerve

## Abstract

**Objectives:**

This study aimed to compare readout-segmented-3, readout-segmented-5, and readout-segmented-7 echo-planar imaging (RS3-EPI, RS5-EPI, and RS7-EPI) of DTI in the assessment of rat sciatic nerve at 3T MR.

**Methods:**

Eight male adult healthy Sprague-Dawley rats were scanned at 3T MR with RS-3 EPI, RS5-EPI, and RS-7 EPI DTI. The image quality of RS-3 EPI, RS-5 EPI, and RS-7 EPI in terms of the nerve morphology, distortions of the nearby femur, muscles, and homogeneity of neuromuscular were evaluated by two experienced radiologists. The correlations between the histopathological and DTI parameters, including fractional anisotropy (FA) and radial diffusivity (RD), were calculated, respectively, and compared in RS-3, RS-5, and RS-7 EPI. The image quality scores for RS-3 EPI, RS-5 EPI, and RS-7 EPI were compared using the Wilcoxon rank-sum test. The correlation between DTI and histopathological parameters was calculated using the Pearson correlation coefficient.

**Results:**

RS-5 EPI yielded the best SNR-values corrected for the acquisition time compared to RS3-EPI and RS7-EPI. The image quality scores of RS-5 EPI were superior to those of RS-3 and RS-7 EPI (*P* = 0.01–0.014) and lower artifacts of the ventral/dorsal margin and femur (*P* = 0.008–0.016) were shown. DTT analysis yielded a significantly higher number of tracts for RS5-EPI compared to RS3-EPI (*P* = 0.007) but no significant difference with RS7-EPI (*P* = 0.071). For the three sequences, FA and RD were well-correlated with the myelin-related histopathological parameters (|r| 0.709–0.965, *P* = 0.001–0.049). The overall correlation coefficients of FA and RD obtained from RS-5 EPI were numerically higher than that with both RS3-EPI and RS7-EPI.

**Conclusion:**

For the rat sciatic nerve DTI imaging, RS-5 EPI offered the best image quality and SNR-values corrected for the acquisition time. The FA and RD derived from the RS-5 EPI were the most sensitive quantitative biomarkers to detect rat sciatic nerve histopathological change.

## Introduction

Diffusion tensor imaging (DTI) has been widely recognized for its value in the central nervous system, such as fiber tracking, assessing fiber continuity, evaluating white matter fiber tract injury, and degeneration of cranial nerve fibers (Mori and Zhang, 2006). DTI technique was subsequently routinely used to determine the integrity of the peripheral nerves, especially in experimental animal studies of nerve injury. Many preclinical and animal studies have proven that DTI is one of the most valuable functional sequences in evaluating peripheral nerve injury repair and function (Guggenberger et al., 2012; Jeon et al., 2018; Zheng et al., 2021). Parameters such as FA and RD derived from DTI are strongly correlated with myelin-related pathological parameters and are considered to be the sensitive biomarkers for detecting early peripheral nerve dysfunction, degeneration, and regeneration (Chen et al., 2017; Farinas et al., 2020; Zheng et al., 2021).

Generally, DTI is performed by using single-shot echo-planar imaging (SS-EPI) sequence for its faster speed. However, this technique is vulnerable to geometric distortion around tissue interface and signal-intensity dropout, primarily because of slow traversal through k-space along the phase-encoding direction (Porter and Heidemann, 2009). Recently, DWI based on readout-segmented echo-planar imaging (RS-EPI), in which the k-space is divided into several segments along the readout direction, has been suggested as an alternative approach to overcome the limitations of the SS-EPI technique (Porter and Heidemann, 2009). Most of the current clinical studies show that RS-EPI has the better image quality and lesion detection, thereby improving the diagnostic performance of DTI (Kida et al., 2016), including in skull base and orbit (Yeom et al., 2013; Chen et al., 2020), breast (Bogner et al., 2012), kidney (Friedli et al., 2017), pelves (Thian et al., 2014), and sacroiliac joint (Zhang et al., 2021). To our knowledge, the application of RS-EPI DWI has not been previously described for the sciatic nerve, especially rat sciatic nerve, which was very small in size and had a relatively high signal in fat set T2WI, presenting challenges in DTI.

Single-shot echo-planar imaging is prone to susceptibility artifacts that manifest as geometric distortion, image blurring, and ghosting artifacts (Drake-Pérez et al., 2018). With the increased numbers of readout segments, although RS-EPI has a more remarkable ability to reduce artifacts and a higher signal-to-noise ratio (SNR), but becomes more vulnerable to motion artifacts and more time-consuming. This pattern is demonstrated in our preliminary study. We found that the image quality of the rat sciatic nerve from DTI with a higher segmented number (e.g., the RS7-EPI) was not better than DTI with a lower segmented number (e.g., the RS3-EPI) and sacrificed more time. In addition, the small and sharp contrast tissue with longer T2 relaxation time, such as nerve tissue, is prone to produce motion-induced deterioration effects (Zaitsev et al., 2015). What’s more, deep and prolonged anesthesia significantly increases the risk of animal death during *in vivo* MRI studies. This further suggests that the image quality does not simply increase with the increasing segmented number but requires a trade-off between the SNR, nerve artifact, and acquisition time. Therefore, the number of readout segments needs to be carefully considered *in vivo* DTI study of rat sciatic nerve. The comparison of the different performance of different readout-segment numbers in RS-EPI DWI has not been previously described for the rat sciatic nerve.

Diffusion tensor imaging parameters such as FA and RD values were considered the sensitive and invasive biomarkers in the monitoring of nerve repair (Chen et al., 2017). However, severe image artifacts and distortion of the nerve can directly affect the region of interest (ROI) drawing and the accuracy of the measured values (Atkinson et al., 2000). In our study, in addition to the evaluation of nerve image quality, we analyzed the correlation between the DTI and pathological parameters related to nerve repair.

Thus, the purposes of the study were, first, to compare the image quality of RS-3 EPI, RS-5 EPI, and RS-7 EPI of DTI of the rat sciatic nerve, and, second, to evaluate the FA and RD values derived from which readout-segment number could provide more accurate pathological information in *in vivo* peripheral nerve monitoring by DTI.

## Materials and Methods

### Subjects

All interventions and animal care procedures were performed by the Guidelines and Policies for Animal Surgery and were approved by the Institutional Animal Use and Care Committee. All animals were obtained from the Animal Experiment Center of Guangdong province. The animals were housed in a standard animal facility with 12-h on/off light conditions and free access to standard food and water. A total of eight male adult healthy Sprague-Dawley rats weighing 250 ± 20 g were used in this study.

### Magnetic Resonance Imaging

All eight healthy adult male Sprague-Dawley rats were anesthetized to deep sleep (7% chloralhydrate, 5 ml/kg, intraperitoneal injection, the rats can deep sleep at least 90 min) and scanned at a 3T scanner (MAGNETOM Prisma, Siemens Healthcare, Erlangen, Germany). After anesthesia, each rat was placed prone in a rat coil (6-cm diameter, eight-channel, Suzhou Medcoil Healthcare Co., Ltd., Suzhou Industrial Park, Suzhou, China) with the limbs fixed with medical adhesive tape to prevent movement further. Both hind limbs were positioned symmetrically.

In our pre-experiments, the acquisition times for readout segment numbers 3, 5, 7, and 9 were 6:12, 10:12, 14:12, and 18:12 min, respectively. To control the overall scan time and reduce the anesthesia risk to a minimum, 18:12 min of readout segment 9 is beyond our tolerance time. Thus, readout segment numbers 3, 5, and 7 were chosen in our study.

**FIGURE 1:**
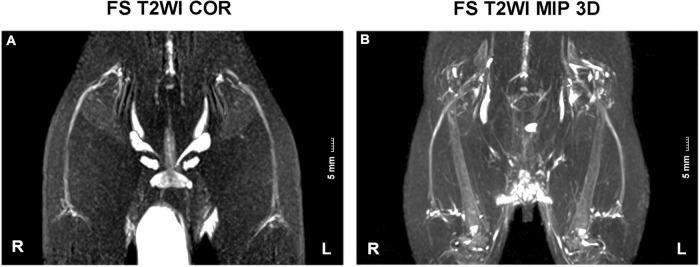
FS T2WI COR and MIP 3D images of sciatic nerves. **(A,B)** T2WI coronal view displayed the morphology of bilateral sciatic nerves. They split above the knee joint into tibial and peroneal nerves. R, right hind limb; L, left hind limb. Scale: 5 mm.

Three axial DTI sequences (RS-3 EPI, RS-5 EPI, and RS-7 EPI) were performed perpendicular to the sciatic nerve with comparable imaging parameters. For the three DTI sequences of RS-3 EPI, RS-5 EPI, and RS-7 EPI: the number of readout segments = 3, 5, and 7, respectively. TE = 75, 63, and 58 ms, respectively; Echo spacing = 0.94, 0.66, and 0.32 ms, respectively. Unified parameters include the following: TR = 4000 ms; slice thickness/gap = 1.5 mm/0 mm; b value = 0, 800 s/mm^2^; diffusion directions = 20; EPI factor = 48; FOV = 70 × 70 mm^2^; Matrix = 100 × 100; No. of sections = 20. A coronal fat-suppressed T2-weighted image was obtained to display the morphology of bilateral sciatic nerves and ensure the correct ROI position for DTI parameters measurement in the sciatic nerve (Figure 1).

### Signal-to-Noise Ratio Measurements

**FIGURE 2:**
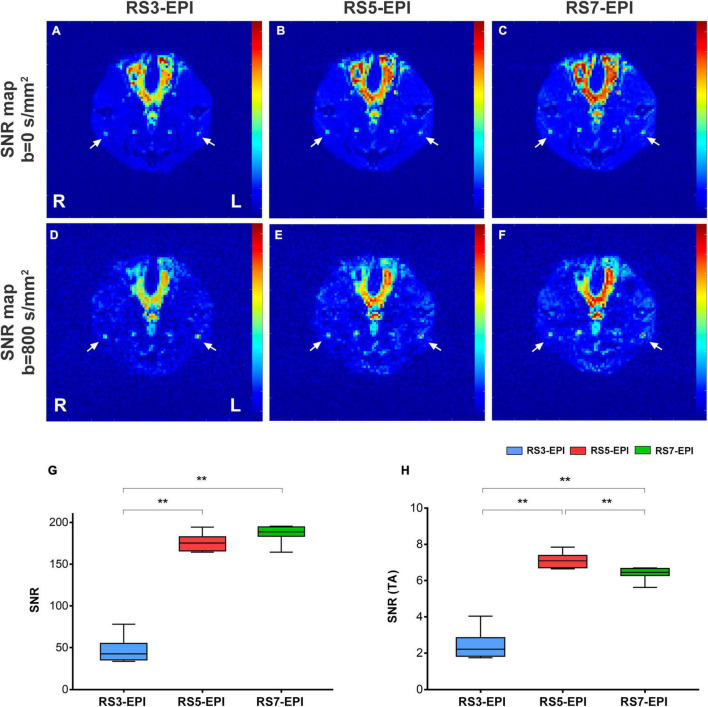
SNR maps **(A–F)**, SNR value **(G)**, and SNR (TA) value **(H)** for RS3-EPI, RS5-EPI, and RS7-EPI. The measured SNR value increases with increasing segmented **(G)** for the three EPI sequences. Regarding SNR-values corrected for the acquisition time, RS5-EPI yielded significantly higher SNR than RS3-EPI and RS7-EPI **(H)**. SNR, signal-to-noise ratio; SNR(TA), SNR Correction due to sequence-specific differences in acquisition time **(H)**. **P ≤ 0.017, *post hoc* analysis with Bonferroni corrected.

The signal-to-noise ratio (SNR) was defined as the ratio between the mean signal intensity of the sciatic nerve (SI_*Nerve*_) and the standard deviation of the background noise (SD_*Background*_) (Figure 2). Using the following equations (Bogner et al., 2012), images and corresponding noise data were post-processed individually (*via* MATLAB MRIqual version 1.2.3), yielding voxel-based SNR maps (Figures 2A–F). Using the following formulas, SNR was calculated from DW images (*b* = 800 s/mm2). ROIs covering each sciatic nerve were manually drawn on the adjacent three slices of the sciatic nerve trunk. The three measurements were averaged, and corresponding SNR values were extracted. The ROI of the background noise was placed in the same phase encoding direction with the sciatic nerve and close to the edge of the image. The size of ROI is uniformly 1 cm^2^.


$$S\,N\,R=\frac{S\,I_{N\,e\,r\,v\,e}}{S\,D_{B\,a\,c\,k\,g\,r\,o\,u\,n\,d}}$$

### Signal-to-Noise Ratio Correction Due to Sequence-Specific Differences in Acquisition Time

To determine the SNR efficiency of a particular acquisition scheme, the measured SNR was divided by the square root of the respective acquisition time (S):


$$S\,N\,R\,\left(T\,A\right)=\frac{S\,N\,R_{N\,e\,r\,v\,e}}{\sqrt{T\,A}}$$

where TA is the total acquisition duration (Manoliu et al., 2017).

### Qualitative Image Evaluation

**FIGURE 3:**
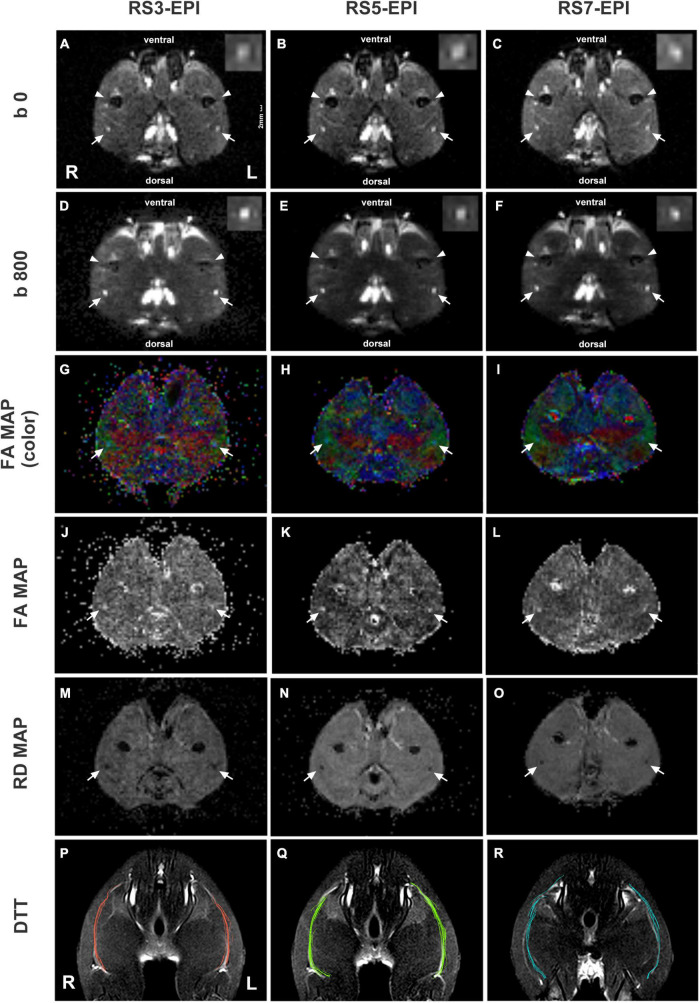
DTI images and DTT of sciatic nerves for RS3-EPI, RS5-EPI, and RS7-EPI. The RS5-EPI images displayed clearer nerve boundaries and minimum nerve distortion, while RS7-EPI showed severe nerve distortion and blurred nerve boundaries **(A–F)**. The FA maps **(G–L)** and RD maps **(M–O)** showed blurred boundaries of RS3-EPI due to poor SNR **(G,J,M)** and of RS5-EPI due to severe nerve distortion **(I,L,O)**. The ventral and dorsal margin artifacts and background noise for RS3-EPI were more pronounced **(D,G,J)**. DTT: RS5-EPI DTI can generate more fibers than RS3-EPI and RS7-EPI and display nerve fibers with a more condensed bundle and realistic architecture **(P–R)**. Conversely, fibers generated by RS3-EPI and RS7-EPI DTI were sparse and discrete, some of which were deviated and in random order. R: right hind limb; L: left hind limb. Scale: 2 mm.

Two independent readers performed image analysis (Reader 1, a radiologist with 10 years of experience in DTI of the nerve; Reader 2, with 4 years of experience in DTI of the nerve). Both readers were blinded to the histopathologic results. The three DTI sequence data were transferred to the workstation (Syngo *Via* 2, Siemens) for image quality evaluation and DTI parameters measurement. DTI images, including *b* = 0, 800 s/mm^2^, FA, and RD maps were generated simultaneously in Neuro 3D modules. The image quality was assessed qualitatively on DWI b = 800 images based on the following factors: sharpness of nerve margin, artifacts of nerve, artifacts of the femur, artifacts of ventral margin, artifacts of dorsal margin, homogeneity of neuromuscular (Figure 3). All images were scored using a 5-point system: 5 means that the images are artifact-free images without distortion and artifact and with great anatomic details of the bilateral sciatic nerve, muscle, and femur; and 1 means impossible to differentiate the anatomic details because of image quality that has been severely distorted by artifacts or low SNR.

### Quantitative Analysis of Diffusion Tensor Imaging Parameters and Diffusion Tensor Tractography

Quantitative analysis of the acquired diffusion data was performed using the syngo.*via* Neuro 3D tool (version 2, Siemens Healthcare). The diffusion tensor parameters (FA and RD) were measured and calculated. ROIs of approximately 3 mm^2^ were manually drawn on the adjacent three slices of the sciatic nerve. The three measurements were averaged for data analysis. Special attention was paid to position the ROIs to minimize the partial volume effect. Transverse DTI images were linked with coronal T2-weighted images to ensure the correct and consistent position of ROIs in the sciatic nerve. Tractography was obtained on the same workstation *via*. A multiple ROI method was used to reconstruct diffusion tensor tractography (DTT). The threshold of FA was set to be 0.15, the maximum fiber angle was 35°, and the minimum fiber length was 15 mm (Chen et al., 2017). The DTT numbers generated by different segmented EPI were calculated and recorded.

### Histopathologic Assessment

Animals were executed after MR imaging by transcardial perfusion with PBS followed by 4% paraformaldehyde in 0.1 M PBS (pH 7.4). The middle stumps of the sciatic nerves were harvested and post-fixed in 4% glutaraldehyde. Transverse semi-thin sections (1 μm thickness) were prepared and stained with toluidine blue to detect nerve myelin. For quantifying toluidine blue staining, sections of the middle stumps were analyzed morphometrically. In brief, an objective magnification of ×1000 was used to take digital images of the entire cross-sectional area of the nerve (60 × 40 μm, 13.7 pixels/μm) on a microscope (Olympus BX60, Japan) for detailed histological quantification. Of these images, five randomly selected measured images (per image area, 240 μm^2^; total area, 1200 μm^2^ of different regions per nerve segment and animal) were analyzed, as previously described. ImageJ software^1^ was used to perform analysis to determine the percentage of axon area (POAA), percentage of myelin area (POMA), thickness of myelin (TOM), and diameter of myelinated fibers (DOMF). The final value used for statistical analysis represents the mean of five measuring images per nerve segment and animal.

### Statistical Analysis

Inter-reader agreement was assessed by using a linear-weighted inter-rater agreement (Kappa) test for the image quality scores at several aspects (sharpness of nerve margin, artifacts of nerve, artifacts of femur, artifacts of ventral muscles, artifacts of dorsal muscles, homogeneity of neuromuscular). The values of Kappa over 0.75, from 0.4 to 0.75, and below 0.4 were regarded as excellent, fair to good, and poor, respectively. Normal distribution of FA, RD, DTT fiber number, SNR, and SNR(TA) was evaluated using the Shapiro–Wilk’s test. To assess potential between-group effects, ANOVA was performed for interval-scaled variables [FA, RD, DTT fiber number, SNR, and SNR(TA)], and Friedman tests were performed for ordinal scaled variables (sharpness of nerve margin, artifacts of nerve, artifacts of femur, artifacts of ventral muscles, artifacts of dorsal muscles, homogeneity of neuromuscular). To evaluate potential differences between RS3-EPI and RS5-EPI, RS3-EPI and RS7-EPI, and RS5-EPI and RS7-EPI, *post hoc* two-sample *t*-tests were performed for interval-scaled variables, and *post hoc* Wilcoxon signed-rank tests were performed for ordinal scaled variables. All statistical tests were Bonferroni corrected for *post hoc* analysis. The degree of association between DTI and histopathological parameters was calculated using the Pearson correlation coefficient. A two-sided *P*-value of 0.05 or less indicated a significant result, and the significance level was *P* ≤ 0.017 for *post hoc* analysis. Statistical analysis was performed by using SPSS (version 23, IBM SPSS, Chicago, IL, United States) and MedCalc (version 19.1.2, MedCalc Software bv, Ostend, Belgium), and plots were created by using GraphPad Prism (version 7) and RStudio (version 1.4.1717).

## Results

### Morphology of Rat Sciatic Nerve

The bilateral sciatic nerve was best displayed on the coronal view of T2WI STIR (Figure 1). They split above the knee joint into tibial and peroneal nerves, and two to three sub-branches can be shown downward. In our study, the thickness of the average rat sciatic nerve trunk ranged from 1 to 2 mm.

### Signal-to-Noise Ratio Analysis

Figure 2 and Table 1 show the SNR and SNR(TA) values for RS3-EPI, RS5-EPI, and RS7-EPI. For the three EPI sequences, Measured SNR increases with increasing segmented. To account for sequence-specific differences in scan duration, correction factors were calculated for the acquisition time. Regarding SNR-values corrected for the acquisition time, RS5-EPI yielded significantly higher SNR than RS3-EPI and RS7-EPI.

**TABLE 1:** SNR and quantitative DTI analysis.

	RS3-EPI	RS5-EPI	RS7-EPI		*P*-value		
					
				ANOVA	RS3 vs. RS5	RS3 vs. RS7	RS5 vs. RS7
SNR	47.106 ± 14.858	175.896 ± 10.563	186.490 ± 10.188	<0.001*	<0.001**	<0.001**	0.093
SNR(TA)	2.443 ± 0.768	7.110 ± 0.425	6.389 ± 0.349	<0.001*	<0.001**	<0.001**	0.015**
FA	0.626 ± 0.007	0.647 ± 0.018	0.607 ± 0.009	<0.001*	0.035	0.001**	0.001**
RD (μm^2^/msec)	0.665 ± 0.021	0.621 ± 0.027	0.693 ± 0.018	<0.001*	0.001**	0.023	<0.001**
DTT fiber number	6.375 ± 4.104	13.375 ± 3.292	8.875 ± 6.266	0.023*	0.007**	0.302	0.071

DTI, diffusion tensor imaging; RS3, No. of readout segments = 3; RS5, No. of readout segments = 5; RS7, No. of readout Segments = 7; SNR, signal-to-noise ratio; SNR(TA), SNR correction due to sequence-specific differences in acquisition time; FA, fractional anisotropy; RD, radial diffusivity; DTT, diffusion tensor tractography; *P ≤ 0.05; **P ≤ 0.017, post hoc analysis with Bonferroni corrected.

### Qualitative Image Evaluation

**FIGURE 4:**
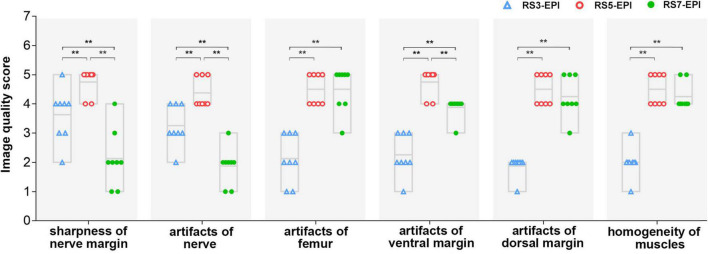
Image quality scores for RS-3 EPI, RS-5 EPI, and RS-7 EPI. The chart showed the image quality scores rated by reader 1 (the more senior radiologist). The image quality scores of RS-5 EPI were superior to those of RS-3 and RS-7 EPI for better display of the nerve and lower artifacts of the ventral/dorsal margin and femur. **P ≤ 0.017, *post hoc* analysis with Bonferroni corrected.

The image quality scores of the three DTI images for the sharpness of nerve margin, artifacts of nerve, artifacts of the femur, artifacts of ventral muscles, artifacts of dorsal muscles, and homogeneity of neuromuscular are shown in Table 2 and Figures 3, 4. The interobserver agreement between the two independent radiologists was excellent (Kappa = 0.753–0.971). Figure 4 shows the image quality scores rated by reader 1 (the more senior radiologist). For reader 1, the Friedman test revealed a significant between-group effect for the image quality scores (all *P* = 0.001). Furthermore, for the sharpness of nerve margin and artifacts of the nerve, the *post hoc* analysis revealed significantly higher image quality scores for RS5-EPI compared to RS3-EPI (*P* = 0.011–0.014) and RS7-EPI (*P* = 0.01–0.011), and significant higher image quality scores for RS3-EPI compared to RS7-EPI (both *P* = 0.016). For artifacts of the ventral muscles, the *post hoc* analysis yielded significantly higher image quality scores for RS5-EPI compared to RS3-EPI (*P* = 0.011) and RS7-EPI (*P* = 0.008) and significant higher image quality scores for RS7-EPI compared to RS3-EPI (*P* = 0.016). For artifacts of the femur, artifacts of dorsal muscles, and homogeneity of the neuromuscular, *post hoc* analysis revealed significantly lower image quality scores for RS3-EPI compared to RS5-EPI (*P* = 0.009–0.01) and RS7-EPI (*P* = 0.008–0.01), and no significant differences were found between RS5-EPI and RS7-EPI (all *P* > 0.017).

**TABLE 2:** Qualitative image evaluation.

Image quality evaluation	RS3 Median (range)	RS5 Median (range)	RS7 Median (range)	*P*-value				Kappa (95% CI) of Reader 1 vs. Reader 2
				
				Friedman	RS3 vs. RS5	RS3 vs. RS7	RS5 vs. RS7	
Reader 1								
Sharpness of the nerve margin	4 (2–5)	5 (4–5)	2 (1–4)	0.001*	0.011**	0.016**	0.011**	0.753 (0.526–0.980)
Artifacts of the nerve	3 (2–4)	4 (4–5)	2 (1–3)	0.001*	0.014**	0.016**	0.010**	0.793 (0.648–0.938)
Artifacts of the femur	2 (1–3)	4.5 (4–5)	5 (3–5)	0.001*	0.009**	0.009**	1.000	0.970 (0.915–1.000)
Artifacts of the ventral margin	2 (1–3)	5 (4–5)	4 (3–4)	0.001*	0.011**	0.016**	0.008**	0.967 (0.903–1.000)
Artifacts of the dorsal margin	2 (1–2)	4.5 (4–5)	4 (3–5)	0.001*	0.009**	0.010**	0.480	0.971 (0.914–1.000)
Homogeneity of the neuromuscular region	2 (1–3)	4.5 (4–5)	4 (4–5)	0.001*	0.010**	0.008**	0.157	0.967 (0.908–1.000)
**Reader 2**								
Sharpness of the nerve margin	4 (2–5)	5 (4–5)	3.5 (2–4)	0.007*	0.020**	0.260	0.017**	\
Artifacts of the nerve	3.5 (2–4)	4 (4–5)	2.5 (1–3)	0.002*	0.038	0.054	0.010**	\
Artifacts of the femur	2 (1–3)	4.5 (4–5)	5 (3–5)	0.001*	0.008**	0.008**	1.000	\
Artifacts of the ventral margin	2 (1–3)	5 (4–5)	4 (3–5)	0.001*	0.011**	0.014**	0.034	\
Artifacts of the dorsal margin	2 (1–2)	4.5 (4–5)	4.5 (3–5)	0.001*	0.009**	0.010**	0.705	\
Homogeneity of the neuromuscular region	2 (2–3)	4.5 (4–5)	4 (4–5)	0.001*	0.009**	0.007**	0.157	\

RS3: No. of readout segments = 3; RS5: No. of readout segments = 5; RS7: No. of readout segments = 7; CI: confidence interval; *P ≤ 0.05; **P ≤ 0.017, post hoc analysis with Bonferroni corrected.

### Quantitative Analysis of Diffusion Tensor Imaging Parameters and Diffusion Tensor Tractography

**FIGURE 5:**
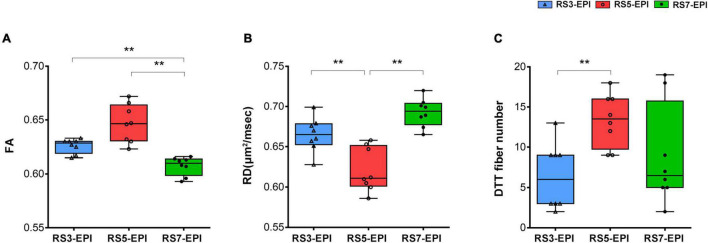
Box plot comparing FA, RD, and DTT fiber numbers for RS3-EPI, RS5-EPI, and RS7-EPI. RS5-EPI yielded higher FA and lower RD than RS3-EPI and RS7-EPI **(A,B)**. The DTT fiber number generated by RS5-EPI was significantly higher than RS3-EPI **(C)**. **P ≤ 0.017, *post hoc* analysis with Bonferroni corrected.

Figure 5 show the FA, RD, and DTT fiber number for RS3-EPI, RS5-EPI, and RS7-EPI. ANOVA yielded a significant group effect for FA (*P* < 0.001), RD (*P* < 0.001), and DTT fiber number (*P* = 0.023). The *post hoc* analysis yielded significantly higher FA for RS5-EPI compared to RS7-EPI (*P* = 0.001) and lower RD for RS5-EPI compared to both RS3-EPI (*P* = 0.001) and RS7-EPI (*P* < 0.001), and higher FA for RS3-EPI compared to RS7-EPI (*P* = 0.001). In addition, DTT analysis yielded a significantly higher number of tracts for RS5-EPI than RS3-EPI (*P* = 0.007) but no significant difference with RS7-EPI (*P* = 0.071).

### Correlation Between Diffusion Tensor Imaging Parameters and Histopathological Parameters

**FIGURE 6:**
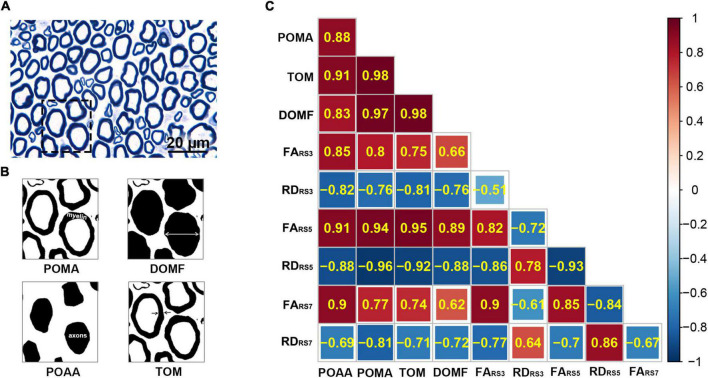
Heatmap of correlation between DTI and histopathological parameters in RS3-EPI, RS5-EPI, and RS7-EPI. **(A)** Correlation coefficients between DTI parameters and histopathological parameters. **(B)** Toluidine blue myelin staining of the sciatic nerves (×1000). Scale: 20 μm. **(C)** Toluidine blue myelin staining was analyzed by ImageJ software to determine the quantitative pathological parameters, including POAA, percentage of axon area; POMA, percentage of myelin area; TOM, the thickness of myelin; DOMF, diameter of myelinated fibers.

Table 3 and Figure 6 show the correlation coefficients between DTI parameters (FA, RD) and histopathological parameters (POAA, POMA, TOM, DOMF) for RS3-EPI, RS5-EPI, and RS7-EPI. For the three sequences, FA was correlated with almost all of the histopathological parameters (*r* = 0.751 to 0.851 for RS3-EPI, *r* = 0.886 to 0.953 for RS5-EPI, and *r* = 0.741 to 0.897 for RS7-EPI, *P* ≤ 0.001–0.036), except the DOMF for both RS3-EPI (*r* = 0.664, *P* = 0.073) and RS7-EPI (*r* = 0.623, *P* = 0.099), and RD was correlated with almost all of the histopathological parameters (*r* = −0.764 to −0.816 for RS3-EPI, *r* = −0.876 to −0.964 for RS5-EPI, and *r* = −0.709 to −0.812 for RS7-EPI, *P* ≤ 0.001−0.049), except the POAA for RS7-EPI (*r* = −0.691, *P* = 0.058). The overall correlation coefficients of FA and RD obtained with RS5-EPI were numerically higher than that with both RS3-EPI and RS7-EPI. The overall correlation coefficients of FA and RD obtained with RS3-EPI were numerically higher than that with RS7-EPI, except for the correlation coefficients between FA and POAA and between RD and POMA.

**TABLE 3:** Correlation coefficients between DTI parameters and histopathological parameters.

	FA			RD		
	RS3-EPI	RS5-EPI	RS7-EPI	RS3-EPI	RS5-EPI	RS7-EPI
POAA	*r* = 0.851 (*P* = 0.007*)	0.911 (0.002*)	0.897 (0.003*)	−0.816 (0.013*)	−0.882 (0.004*)	−0.691 (0.058)
POMA	0.799 (0.017*)	0.944 (<0.001*)	0.769 (0.026*)	−0.764 (0.027*)	−0.964 (<0.001*)	−0.812 (0.014*)
TOM	0.751 (0.032*)	0.953 (<0.001*)	0.741 (0.036*)	−0.808 (0.015*)	−0.922 (<0.001*)	−0.709 (0.049*)
DOMF	0.664 (0.073)	0.886 (0.003*)	0.623 (0.099)	−0.765 (0.027*)	−0.876 (0.004*)	−0.716 (0.046*)

DTI, diffusion tensor imaging; FA, fractional anisotropy; RD, radial diffusivity. Histopathological parameters: POAA, percentage of axon area; POMA, percentage of myelin area; TOM, thickness of myelin; DOMF, diameter of myelinated fiber. r value, correlation coefficient value. P-value in parentheses, the result of correlation. *P ≤ 0.05.

## Discussion

To evaluate the feasibility of segment-number 3, 5, and 7 of RS-EPI for DWI/DTI of rat sciatic nerve, we assessed qualitative data using image quality scoring in several detailed aspects on axial DWI images, and quantitative data using SNR, SNR(TA), number of tracts, FA, and RD value together with their correlation with pathologic parameters for each sciatic nerve in eight healthy rats. We found that referring to the rat sciatic nerve, RS5-EPI yielded optimal image quality, SNR(TA), pathology-correlated FA and RD value, and the richest number of fiber tracts compared to the RS7-EPI as well as RS3-EPI. Therefore, the current results demonstrate that DWI/DTI imaging of the rat sciatic nerve, RS5-EPI, is feasible, and the DTI parameters derived from RS5-EPI showed a stronger linear correlation with histopathological parameters.

In our study, the SNR tended to increase with an increased segment number. The SNR for RS5-EPI and RS7-EPI were significantly higher than SNR for RS3-EPI. This difference may be because RS-EPI samples subsets of k-space points in the readout direction in each shot, thereby permitting a substantial reduction in echo spacing, TE, and T2* signal blurring. Multishot approaches typically can accommodate higher spatial resolution and possess higher SNR (Porter and Heidemann, 2009). As the increases of segment number, the acquisition time increases significantly (Atkinson et al., 2000). In our study, although RS7-EPI yielded the highest SNR originally, regarding SNR-values corrected for the acquisition time, RS5-EPI yielded higher SNR(TA) than both RS3-EPI and RS7-EPI. This shows that the SNRs of RS5-EPI and RS7-EPI are very close, but RS5-EPI requires a lower time cost and has a better application value.

In our study, the RS5-EPI sequence showed better image quality of the rat sciatic nerve compared with both RS3-EPI and RS7-EPI, displaying clearer margin and fewer artifacts of the rat sciatic nerve. RS5-EPI performed better than RS3-EPI because RS-EPI samples subsets of k-space points in the readout direction in each shot, and the resulting shorter echo spacing reduces susceptibility artifacts, geometric distortion, and T2* blurring by accelerating the k-space traversal along the direction of the readout. At the same time, the SNR has been improved. RS-EPI incorporated a 2D navigator-based reacquisition technique, which corrects motion-induced phase errors (Zaitsev et al., 2015). Unexpectedly, although RS7-EPI had the most subsets, the longest acquisition time, and the highest SNR, it yielded the lowest image quality scores of the rat sciatic nerve of the three RS-EPI. The most important reasons are that although multi-shot techniques acquire only a segment of k-space after each diffusion sensitization and enable higher resolution and reduced susceptibility artifacts. Motion during the diffusion sensitization leads to phase changes and k-space offsets. These differ between segments in multi-shot sequences, which can lead to severe image artifacts if uncorrected (Atkinson et al., 2000). Motion as a function of the k-space position is the most relevant parameter for the appearance of artifacts. In the case of interleaved multishot k-space acquisitions, such as the RS-EPI DTI, even slow continuous drifts produce significant ghosting; for example, the gradual relaxation of the neck muscles in head imaging or of the muscles around the sciatic nerve after anesthesia in our study (Zaitsev et al., 2015). With the increase of readout segments, RS-EPI becomes more vulnerable to motion artifacts. To reduce these motion artifacts, a nonlinear phase correction and reacquisition technique based on navigation echoes is used in RS-EPI (Nguyen et al., 1998; Porter and Mueller, 2004). However, the correction would fail for severely corrupted data sets since the reacquisition is time-limited (only 20% of the standard measurement time) (Porter and Heidemann, 2009). Meanwhile, with the increase of segments, the scanning time of RS-EPI increases multiply (Friedli et al., 2017; McKay et al., 2020), and the corrupted data due to motion increases either. It would be difficult to reacquire all the corrupted data in a limited time, resulting in partial data loss. This explains the heavier motion artifacts and geometric distortion of the RS-7 EPI than the RS-5 EPI. The corrupted data was still in time to be corrected with a high probability. What’s more, it is essential wherein k-space fast motion occurs, as data corruption near the center of the k-space produces stronger artifacts than data corruption near the k-space periphery (Zaitsev et al., 2015). This is why the RS7-EPI scores in all aspects were more discrete than in the other two sequences in our study. When the motion occurs near the k-space periphery, the correction would be successful; while the motion occurs near the k-space center, the correction would be failed. In our study, due to the small size of the rat coil and the smaller voxel size with higher resolution, the signal strength is stronger, and the motion artifacts are more substantial (Gruber et al., 2018; Vergara Gomez et al., 2019). In addition to the extreme sensitivity to motion, the unsatisfied nerve image quality of RS7-EPI resulted from some other reasons. The sharp contrast tissue with a longer T2 relaxation time, such as nerve tissue, can produce motion-induced deterioration effects, which appear as a blurred border or geometric distortion (Chavhan et al., 2014). Conversely, the bone cortex and muscles with shorter T2 relaxation time have stronger resistance to motion artifacts than neural tissues. This explains why for the RS7-EPI, which is most sensitive to motion artifacts, the image quality of femoral and muscle scored excellent while the sciatic nerve scored poorly.

In our study, in terms of the artifacts of ventral margin and dorsal margin, both RS5-EPI and RS7-EPI performed very well. This is because in areas with more susceptibility artifacts, such as the ventral and dorsal margin (adjacent to the air), the readout segmented EPI becomes very dominant in reducing geometric distortion caused by susceptibility artifacts (Bogner et al., 2012; Yeom et al., 2013; Zaitsev et al., 2015). Moreover, we found that the performance of RS7-EPI at the ventral margin was not as good as that at the dorsal border, which may be due to the presence of the scrotum, bladder, and intestine, which are water-containing organs on the ventral side, indicating that RS7-EPI is more prone to motion artifacts for tissues with high T2, which is also consistent with the fact that neural tissues with high T2 signal are more prone to motion artifacts at RS7-EPI (Chavhan et al., 2014). The superior homogeneity of the muscles yielded by RS5-EPI and RS7-EPI due to their excellent SNR compared with RS3-EPI. In our study, RS-5 EPI generated more DTT fiber numbers and displayed a more condensed bundle and realistic architecture. This is first due to the superior SNR of RS5-EPI and second related to the stronger resistance to motion artifact.

In summary, RS5-EPI not only has the best SNR (TA) and best resists motion-induced artifacts and nerve display but also has fewer artifacts in the peripheral edge region and shows good muscle homogeneity, allowing the best image quality to be acquired in the DTI scan of the rat sciatic nerve.

Diffusion tensor imaging offers quantitative information on the structure and orientation features to assess peripheral nerve diseases. Among these DTI metrics, FA and RD were thought to be the most stable and sensitive biomarkers for evaluating peripheral nerve regeneration (Naraghi et al., 2015; Chen et al., 2017). FA can reflect the packing density of axons within a voxel. At the same time, RD quantifies the diffusion perpendicular to the axonal orientation and is supposed to reflect myelin-related information (Jeon et al., 2018). Previously, peripheral nerve repair-related studies have shown that in the process of nerve fiber regeneration, with the density and integrality of myelin increases, FA value increases while RD value decreases (Chen et al., 2017; Farinas et al., 2020; Zheng et al., 2021). Similarly, in the peripheral nerve degeneration process of healthy aging people, nerve anisotropy (FA) decreases and RD increases with the decrease of the number of myelinated fibers (Jeon et al., 2018). Therefore, FA and RD value are sensitive biomarkers to detect minor myelin. In our study, the overall correlation coefficients of FA and RD obtained with RS5-EPI were numerically higher than that with both RS3-EPI and RS7-EPI. The FA and RD values for the three RS EPI were correlated differently with almost all the myelin-related pathological parameters of the rat sciatic nerve. Therefore, this indicated that referring to rat sciatic nerve, RS-5 EPI can acquire better image quality and provide more sensitive and accurate histological information compared to RS3-EPI and RS7-EchangesPI.

Our study had limitations. First, healthy adult rats were used in this study. The differences in DTI parameters for monitoring pathological changes in the sciatic nerve of healthy rats were minor. Future comparative studies on the rat sciatic nerve injury model will be more meaningful. Second, we did not include the comparison of single short EPI in our research. Despite the severe magnetic sensitivity artifacts at the edges of EPI, the single short EPI might yield good image quality of the nerve because of the longer TE, stronger resistance to motion artifacts, and shorter acquisition time. It would be interesting to compare RS-EPI and SS-EPI in the sciatic nerve in the future.

In conclusion, for the rat sciatic nerve DTI imaging, the RS-5 EPI sequence was more robust to motion artifacts and offered a shorter scanning time than the RS-7 EPI sequence. Regarding SNR-values corrected for the acquisition time, RS5-EPI yielded significantly higher SNR than RS3-EPI and RS7-EPI. The FA and RD derived from the RS-5 EPI sequence might be highly sensitive quantitative biomarkers to detect rat sciatic nerve histopathological change. These findings will also inform the DTI sequence optimization in MRI basic research for peripheral nerves.

## Data Availability Statement

The original contributions presented in this study are included in the article/supplementary material, further inquiries can be directed to the corresponding authors.

## Ethics Statement

The animal study was reviewed and approved by the Institutional Animal Care and Use Committee of Jennio Biotech Co., Ltd. Written informed consent was obtained from the owners for the participation of their animals in this study.

## Author Contributions

YC: data collection and analysis, experiment design, and writing of the manuscript. ZP: statistical analysis of data and creation of statistical charts. FM: rat MRI scan, sequence optimization, and technical assistance. QX: pathological staining and analysis. LH: image processing. XP: MRI data acquisition. XY: pathological staining. YW: discussion and revision of the manuscript. XL and HL: full access to all the data in the study and responsible for the integrity of the data and the accuracy of the data analysis. All authors contributed to the article and approved the submitted version.

## Conflict of Interest

YW was employed by Siemens Healthcare (China). The remaining authors declare that the research was conducted in the absence of any commercial or financial relationships that could be construed as a potential conflict of interest.

## Publisher’s Note

All claims expressed in this article are solely those of the authors and do not necessarily represent those of their affiliated organizations, or those of the publisher, the editors and the reviewers. Any product that may be evaluated in this article, or claim that may be made by its manufacturer, is not guaranteed or endorsed by the publisher.

## Footnotes

1
https://imagej.net

## Abbreviations

DTI: diffusion tensor imaging
FA: fractional anisotropy
RD: radial diffusivity
POAA: percentage of axon area
POMA: percentage of myelin area
TOM: the thickness of myelin
DOMF: diameter of myelinated fibers
SS: single-shot
RS: readout-segmented
EPI: echo-planar imaging
ROI: region of interest
SI: signal intensity
SD: standard deviation
SNR: signal-to-noise ratio
ICC: intraclass correlation coefficient.
